# Reducing Visceral-Motion-Related Artifacts on the Liver with Dual-Energy CT: A Comparison of Four Different CT Scanner Techniques

**DOI:** 10.3390/diagnostics12092155

**Published:** 2022-09-05

**Authors:** Sergio Grosu, Korawan Vijittrakarnrung, Zhen J. Wang, Markus M. Obmann, Yuxin Sun, Mark D. Sugi, Benjamin M. Yeh

**Affiliations:** 1 Department of Radiology and Biomedical Imaging, University of California, San Francisco, 513 Parnassus Ave, San Francisco, CA 94143, USA; 2 Department of Radiology, University Hospital, LMU Munich, Marchioninistr 15, 81377 Munich, Germany; 3 Department of Radiology and Nuclear Imaging, University Hospital Basel, Petersgraben 4, CH-4051 Basel, Switzerland

**Keywords:** tomography, X-ray computed, dual-energy computed tomography, abdomen, liver, artifacts, peristalsis

## Abstract

*Purpose*: To assess the influence of different dual-energy CT (DECT) scanner techniques on the severity of visceral-motion-related artifacts on the liver. *Methods*: Two independent readers retrospectively evaluated visceral-motion-related artifacts on the liver on 120-kVp(-like), monoenergetic low- and high-keV, virtual non-contrast (VNC), and iodine images acquired on a dual-source, twin-beam, fast kV-switching, and dual-layer spectral detector scanner. Quantitative assessment: Depth of artifact extension into the liver, measurements of Hounsfield Units (HU) and iodine concentrations. Qualitative assessment: Five-point Likert scale (1 = none to 5 = severe). Artifact severity between image reconstructions were compared by Wilcoxon signed-rank and paired t-tests. *Results*: 615 contrast-enhanced routine clinical DECT scans of the abdomen were evaluated in 458 consecutive patients (mean age: 61 ± 14 years, 331 men). For dual-source and twin-beam scanners, depth of extension of artifacts into the liver was significantly shorter and artifact severity scores significantly lower for 120-kVp-like images compared with the other image reconstructions (*p* < 0.001, each). For fast kV-switching and spectral detector scanner images, depth of extension of artifacts was significantly shorter and artifact severity scores significantly lower for iodine images (*p* < 0.001, each). Dual-source 120-kVp-like and spectral detector iodine images reduced artifacts to an extent that no significant difference in HU or iodine concentrations between artifacts (dual-source: 97 HU, spectral detector: 1.9 mg/mL) and unaffected liver parenchyma (dual-source: 108 HU, spectral detector: 2.1 mg/mL) was measurable (dual-source: *p* = 0.32, spectral detector: *p* = 0.15). *Conclusion*: Visceral-motion-related artifacts on the liver can be markedly reduced by viewing 120-kVp-like images for dual-source and twin-beam DECT scanners and iodine images for fast kV-switching and dual-layer spectral detector DECT scanners.

## 1. Introduction

Computed tomography (CT) is frequently used for oncologic staging and follow-up. Optimization of CT image quality is essential for accurate assessment of the liver, as it is commonly affected by metastases and primary tumors that may be small or subtle. Image artifacts at CT degrade image quality and may compromise diagnostic accuracy [1,2].

Different types of CT image artifacts are known to relate to CT data acquisition, image reconstruction, metallic implants, and patient motion [1,2,3,4,5]. In particular, motion-related artifacts caused by involuntary movement such as intestinal peristalsis or cardiac motion are challenging. The peristalsis-related movement of intraluminal gas in the stomach or bowel during CT scanning can cause bright and dark streak artifacts in CT images [2,6,7,8]. Due to the anatomical proximity of the liver to the stomach, the liver, particularly the left liver lobe, is frequently affected by visceral-motion-related artifacts [7,9].

Advances in CT techniques such as rapid scanning and software-based correction algorithms can reduce the severity and frequency of visceral-motion-related artifacts [2,10,11]. However, with a prevalence ranging from 25% to 70%, these artifacts remain frequent in CT scans of the abdomen, often affecting the liver [2,7,9].

Another method to reduce visceral-motion-related artifacts is enabled by dual-energy computed tomography (DECT) [7]. DECT utilizes the imaging data from two different X-ray photon energy levels, typically to identify and quantify material composition [12,13,14,15,16,17]. Different scanner implementations are currently used in clinical practice to acquire low- and high-kVp datasets in order to generate DECT images: Dual-source DECT scanners use two X-ray tubes at different tube voltages and two detector arrays mounted orthogonally in the gantry [12,18,19]. Twin-beam DECT scanners use a gold/tin split filter applied to a single X-ray tube to create a high-energy spectrum in one half and a low-energy spectrum in the other half of the single beam [12,13,18]. Fast kV-switching DECT scanners use a single X-ray tube that switches rapidly between a high- and low-energy spectrum [12,18,19]. Dual-layer spectral detector DECT scanners use a conventional polychromatic X-ray beam that is separated into low- and high-energy photons at the detector level [12,18,19].

Low-keV (40–50 keV) monoenergetic and iodine DECT image reconstructions improve liver lesion conspicuity [20,21,22,23]. Furthermore, iodine DECT image reconstructions are helpful in the characterization of liver lesions [24,25]. For dual-layer spectral detector DECT scanners, it was shown that visceral-motion-related artifacts on the liver can be substantially reduced by viewing iodine DECT image reconstructions [9]. However, as DECT methodologies differ significantly between DECT scanners, it is unclear whether iodine images are useful for visceral-motion-related artifact reduction in other scanner implementations.

The aim of our study was to assess the influence of clinical dual-source, twin-beam, fast kV-switching, and dual-layer spectral detector DECT image reconstructions on the severity of visceral-motion-related artifacts on the liver.

## 2. Materials and Methods

In total, 131 of the 458 patients were included in a previous study [9]. This prior article dealt with visceral-motion-related artifact reduction with a dual-layer spectral detector DECT scanner only, whereas in this manuscript, we report on visceral-motion-related artifact reduction with different DECT scanner models, as DECT methodologies differ significantly between models.

This study is compliant with the Health Insurance Portability and Accountability Act. Need for informed consent was waived by the institutional review board. The National Institutes of Health and Philips Healthcare provided funding for our study as part of a research grant. The funders had no role in the study design, data collection, data analyses, data interpretation, manuscript writing, or in publishing the results.

### 2.1. Study Population

In this retrospective analysis, we evaluated all contrast-enhanced CT scans (arterial phase, venous phase, delayed phase) of the abdomen acquired as part of clinical routine on a dual-source CT scanner during the time period from 29 April 2021 to 28 May 2021, on a twin-beam CT scanner from 8 May 2021 to 28 May 2021, on a fast kV-switching CT scanner from 1 February 2018 to 5 March 2018, and on a dual-layer spectral detector CT scanner from 13 September 2017 to 1 April 2018. Patients <18 years-of-age were excluded. No antispasmodic medication was administered. Following the clinical routine standard operating procedure of our department, fasting prior to CT image acquisition was not required. CT scans with artifacts on the liver originating from oral contrast material or metallic foreign materials were excluded. CT scans with missing source dual-energy data were excluded from artifact evaluation.

### 2.2. CT Image Acquisition

DECT image data sets were acquired on four different scanners: (1) Dual-source CT scanner (Somatom Definition Flash; Siemens Healthineers, Forchheim, Germany), (2) Twin-beam CT scanner (Somatom Definition Edge; Siemens Healthineers, Forchheim, Germany), (3) Fast kV-switching CT scanner (Revolution; GE Healthcare, Chicago, Illinois), (4) Dual-layer spectral detector CT scanner (IQon; Philips Healthcare, Cleveland, OH, USA). Further details are provided in Table 1 and in the Supplementary Material.

### 2.3. Image Analysis

Images were viewed on standard picture archiving and communication system (PACS) workstations. The commercially available dedicated postprocessing software Syngo. Via version VB30A (Siemens Healthineers, Forchheim, Germany) was used for the evaluation of dual-source and twin-beam images, GSI Volume Viewer version 13.0 (GE Healthcare, Chicago, IL, USA) for fast kV-switching images, and IntelliSpace Portal version 11.1 (Philips Healthcare, Cleveland, OH, USA) for dual-layer spectral detector images. Two independent radiologists (Reader A: S.G., 4 years of experience in abdominal radiology) (Reader B: K.V., 6 years of experience in abdominal radiology) analyzed axial images of the different DECT reconstructions. DECT images were reviewed on default window level settings for the presence or absence of visceral-motion-related artifacts on the liver. The organ where the visceral-motion-related artifacts originated from was recorded. An artifact was considered to be present if it was scored as visible by one or both readers. To assess inter-reader and intra-reader variability, all CT scans with visceral-motion-related artifacts were evaluated independently by Reader A and B, then 26 CT scans with visceral-motion-related artifacts were evaluated again four weeks after the first reading by Reader A.

### 2.4. Quantitative Visceral-Motion-Related Artifact Evaluation

The depth of artifact extension into the liver parenchyma was measured from the affected liver capsule to the last visible extent of the artifact on the liver parenchyma in millimeters on axial 120-kVp(-like) images, monoenergetic low-keV (dual-source scanner: 40-keV, twin-beam scanner: 40-keV, fast kV-switching scanner: 40-keV, dual-layer spectral detector scanner: 40-keV), monoenergetic high-keV (dual-source scanner: 190-keV, twin-beam scanner: 190-keV, fast kV-switching scanner: 140-keV, dual-layer spectral detector scanner: 200-keV), virtual non-contrast (VNC), and iodine images.

Circular regions of interest (ROI) were manually placed in the brightest area of the artifact, darkest area of the artifact, and in neighboring unaffected liver parenchyma. ROImax was defined as bright, ROImin as dark, on low-keV images. Areas of inhomogeneity due to partial volume effect, vessels, or tissue borders were avoided. ROIs were propagated onto the axial 120-kVp(-like), low-keV, high-keV, VNC, and iodine images with identical ROI sizes in identical anatomical ROI locations. ROI measurements are presented in HU (120-kVp(-like), low-keV, high-keV, and VNC images) or iodine concentrations in mg/mL (iodine images). Comparisons of ROI measurements were performed between artifacts and neighboring liver parenchyma not affected by artifacts, which served as reference tissue.

### 2.5. Qualitative Visceral-Motion-Related Artifact Evaluation

Visceral-motion-related artifact severity on the liver in 120-kVp(-like), low- and high-keV, VNC, and iodine images was qualitatively evaluated on a five-point Likert scale [9]: 1 = Absence of visceral-motion-related artifact on the liver; 2 = Visible visceral-motion-related artifact with no effect on diagnosis on the liver; 3 = Moderate visceral-motion-related artifact that may decrease confidence in diagnosing a 0.5 to 0.9 cm liver lesion; 4 = Distinct visceral-motion-related artifact that prevents the diagnosis of a 0.5 to 0.9 cm liver lesion, and that may decrease confidence in diagnosing a ≥ 1.0 cm liver lesion; 5 = Severe visceral-motion-related artifact that prevents the diagnosis of a ≥ 1.0 cm liver lesion [9].

### 2.6. Statistical Analysis

Continuous variables were expressed as mean ± standard deviation (SD), categorical variables as frequencies and percentages. The Shapiro–Wilk test was used for testing for normality.

Intra-reader and inter-reader agreement for qualitative artifact scores and quantitative artifact measurements was calculated using intraclass correlation coefficients (ICC) and weighted Cohen’s kappa coefficients, respectively [26,27,28].

The Wilcoxon signed-rank test was used to compare the depth of visceral-motion-related artifact extension into the liver and qualitative artifact scores between 120-kVp(-like), low-keV, high-keV, VNC, and iodine images of each scanner, respectively. The paired t-test was performed to evaluate the relationship between ROI measurements (HU, iodine concentration) of visceral-motion-related artifacts and the neighboring liver parenchyma not affected by artifacts in 120-kVp(-like), low-keV, high-keV, VNC, and iodine images of each scanner, respectively.

In addition, *p*-values < 0.05 were considered to denote statistical significance. Statistical analysis was performed with the open-source software RStudio Version 1.4.1103 (RStudio Team (2020), RStudio: Integrated Development for R. RStudio, PBC, Boston, MA, USA).

## 3. Results

### 3.1. Study Population

In total, we evaluated 615 contrast-enhanced CT scans of the abdomen in 458 patients (mean age: 61 ± 14 years, 331 men) (dual-source scanner: 127 scans in 90 consecutive patients, twin-beam scanner: 142 scans in 140 consecutive patients, fast kV-switching scanner: 126 scans in 97 consecutive patients, dual-layer spectral detector scanner: 220 scans in 131 consecutive patients). A total of 36/615 (6%) CT scans were excluded due to artifacts on the liver originating from metallic foreign materials or oral contrast material.

**Figure 1:**
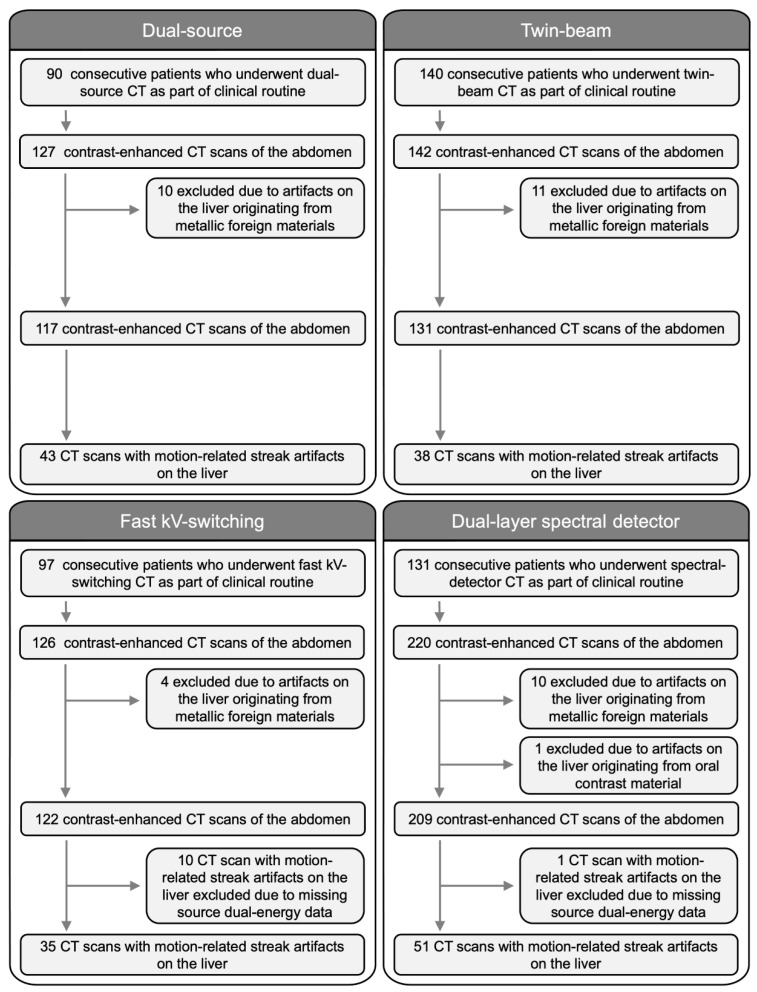
Flow chart of the study population. In total 615 contrast-enhanced CT scans of the abdomen in 458 patients (mean age: 61 ± 14 years, 331 men) were evaluated. Dual-source: 90 patients (mean age: 58 ± 14 years, 50 men). Twin-beam: 140 patients (mean age: 51 ± 19 years, 72 men). Fast kV-switching: 97 patients (mean age: 66 ± 11 years, 89 men). Dual-layer spectral detector: 131 patients (mean age: 68 ± 10 years, 120 men).

Visceral-motion-related artifacts on the liver were present in 178/579 (31%) CT scans. Artifacts involved the left liver lobe only in 169/178 (95%), the right liver lobe only in 3/178 (2%), and both liver lobes in 6/178 (3%) CT scans. Visceral-motion-related artifact origin was the stomach in 152/178 (85%), the transverse colon in 17/178 (10%), and the heart in 9/178 (5%) CT scans. Further details are provided in Figure 1 and in the Supplementary Material.

### 3.2. Quantitative Visceral-Motion-Related Artifact Evaluation

The inter-reader agreement in the quantitative assessment of depth of artifact extension into the liver was moderate (ICC = 0.56, *p* < 0.001), and of Hounsfield Unit and iodine concentration differences of visceral-motion-related artifacts from normal liver parenchyma was moderate (ICC = 0.64, *p* < 0.001). The intra-reader agreement in the assessment of depth of artifact extension was excellent (ICC = 0.97, *p* < 0.001), and of Hounsfield Unit and iodine concentration differences was excellent (ICC = 0.98, *p* < 0.001).

**Figure 2:**
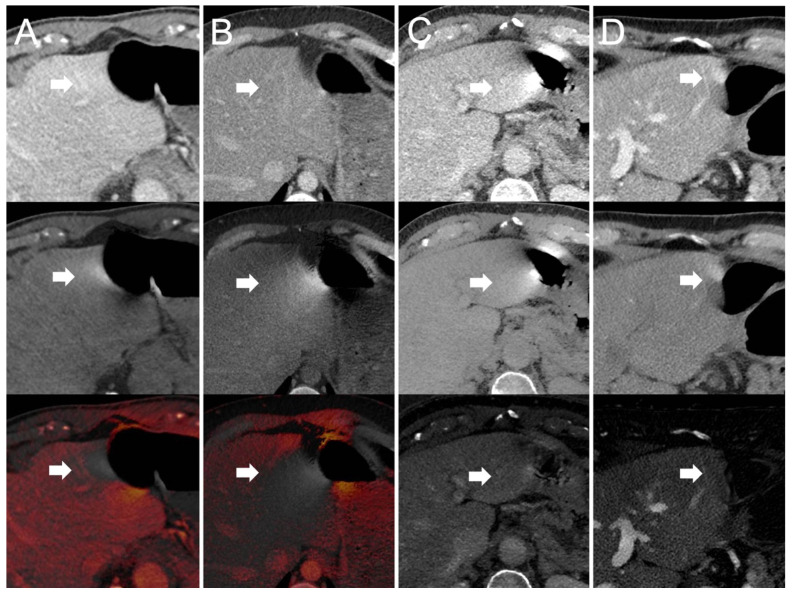
120-kVp(-like) (top row), VNC (middle row), and iodine images (bottom row) acquired on a (**A**) dual-source, (**B**) twin-beam, (**C**) fast kV-switching, and (**D**) dual-layer spectral detector DECT scanner. Visceral-motion-related artifacts on the liver (arrows) in VNC (middle row) and iodine images (bottom row) acquired on a (**A**) dual-source DECT scanner and (**B**) twin-beam DECT scanner are substantially reduced in 120-kVp-like images (top row). Visceral-motion-related artifacts (arrows) in 120-kVp(-like) (top row) and VNC images (middle row) acquired on a (**C**) fast kV-switching DECT scanner and (**D**) dual-layer spectral detector DECT scanner are substantially reduced in iodine images (bottom row).

**Figure 3:**
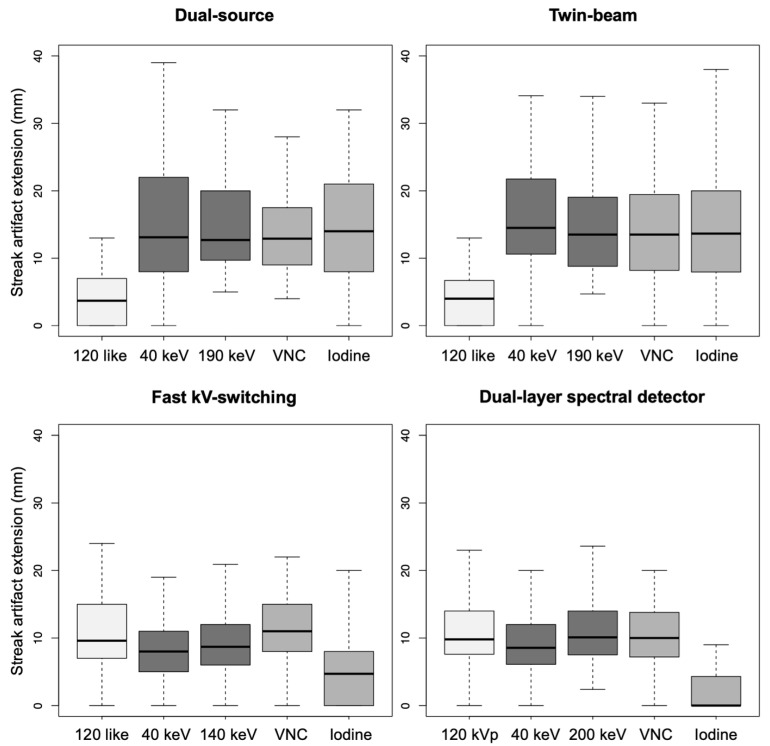
Depth of extension of visceral-motion-related artifacts into the liver parenchyma measured in axial 120-kVp(-like), low- and high-keV, VNC, and iodine images acquired on a dual-source, twin-beam, fast kV-switching, and dual-layer spectral detector CT scanner.

(1) Dual-source scanner: Depth of extension of visceral-motion-related artifacts into the liver (see Figure 2 and Figure 3) was significantly shorter (*p* < 0.001, each) for 120-kVp-like images (mean length: 5 ± 6 mm) compared with 40-keV (mean length: 16 ± 11 mm), 190-keV (mean length: 16 ± 9 mm), VNC (mean length: 15 ± 10 mm), and iodine (mean length: 16 ± 11 mm) images. Mean ROI measurements (HU or iodine concentration, respectively) were significantly different in the bright and dark artifact components compared to the neighboring liver parenchyma not affected by artifacts in 40-keV (*p* < 0.001, each), 190-keV (*p* < 0.001, each), VNC (*p* < 0.001, each), and iodine images (*p* < 0.001, each). However, in 120-kVp-like images, mean ROI measurements were not significantly different in bright (ROImin) artifact components compared to unaffected liver parenchyma (*p* = 0.32), as opposed to dark (ROImax) artifact components (*p* < 0.001).
(2) Twin-beam scanner: Depth of extension of visceral-motion-related artifacts into the liver was significantly shorter (*p* < 0.001, each) for 120-kVp-like images (mean length: 4 ± 5 mm) compared with 40-keV (mean length: 18 ± 12 mm), 190-keV (mean length: 16 ± 11 mm), VNC (mean length: 15 ± 11 mm), and iodine (mean length: 16 ± 11 mm) images. Mean ROI measurements were significantly different in the bright and dark artifact components compared to unaffected liver parenchyma in 120-kVp-like (*p* = 0.006, *p* = 0.03), 40-keV (*p* < 0.001, each), 190-keV (*p* < 0.001, each), VNC (*p* < 0.001, each), and iodine images (*p* < 0.001, each).
(3) Fast kV-switching scanner: Depth of extension of visceral-motion-related artifacts into the liver was significantly shorter (*p* < 0.001, each) for iodine (mean length: 6 ± 7 mm) images compared with 120-kVp-like (mean length: 11 ± 7 mm), 40-keV (mean length: 9 ± 8 mm), 140-keV (mean length: 10 ± 8 mm), and VNC (mean length: 13 ± 8 mm) images. Mean ROI measurements were significantly different in the bright and dark artifact components compared to unaffected liver parenchyma in 120-kVp-like (*p* < 0.001, each), 40-keV (*p* < 0.001, each), 140-keV (*p* < 0.001, each), VNC (*p* < 0.001, each), and iodine images (*p* < 0.001, each).
(4) Dual-layer spectral detector scanner: Depth of extension of visceral-motion-related artifacts into the liver was significantly shorter (*p* < 0.001, each) for iodine (mean length: 2 ± 5 mm) images compared with 120-kVp (mean length: 11 ± 5 mm), 40-keV (mean length: 10 ± 6 mm), 200-keV (mean length: 11 ± 5 mm), and VNC (mean length: 11 ± 5 mm) images. Mean ROI measurements were significantly different in the bright and dark artifact components compared to unaffected liver parenchyma in 120-kVp (*p* < 0.001, each), 40-keV (*p* < 0.001, each), 200-keV (*p* < 0.001, each), and VNC (*p* < 0.001, each) images. However, in iodine images mean ROI measurements were not significantly different in bright (ROImax) artifact components compared to unaffected liver parenchyma (*p* = 0.15), as opposed to dark (ROImin) artifact components (*p* < 0.001). Further details on quantitative artifact measurements are provided in Table 2 and the Supplementary Material.

### 3.3. Qualitative Visceral-Motion-Related Artifact Evaluation

The inter-reader agreement in the qualitative evaluation (five-point Likert scale) of visceral-motion-related artifact severity on the liver was substantial (kappa coefficient = 0.64, *p* < 0.001) (dual-source scanner: kappa coefficient = 0.70, *p* < 0.001; twin-beam scanner: kappa coefficient = 0.65, *p* < 0.001; fast kV-switching scanner: kappa coefficient = 0.60, *p* < 0.001; dual-layer spectral detector scanner: kappa coefficient = 0.51, *p* < 0.001). The intra-reader agreement was almost perfect (kappa coefficient = 0.89, *p* < 0.001).

**Figure 4:**
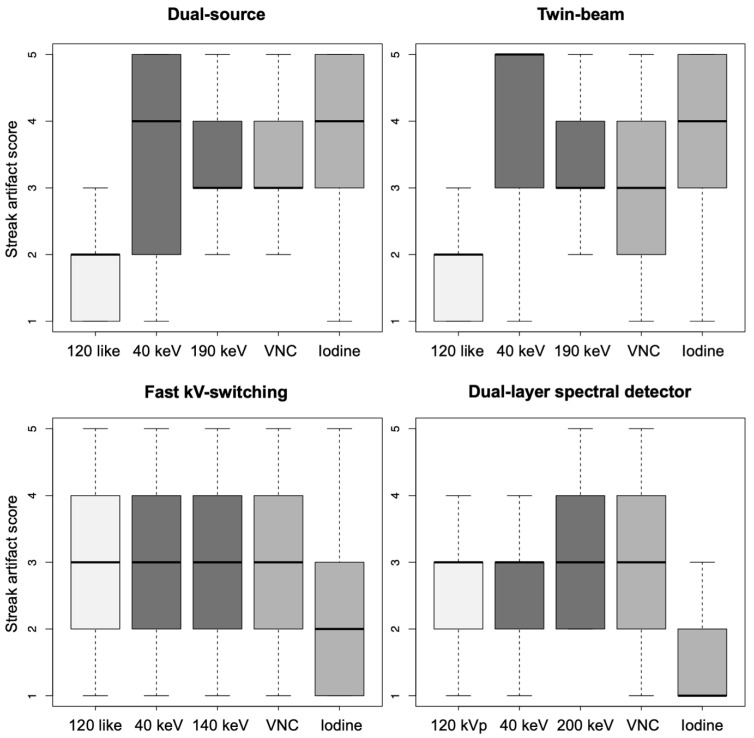
Qualitative artifact scores (1 = none to 5 = severe) of visceral-motion-related artifacts on the liver in axial 120-kVp(-like), low- and high-keV, VNC, and iodine images acquired on a dual-source, twin-beam, fast kV-switching, and dual-layer spectral detector CT scanner.

(1) Dual-source scanner: Qualitative artifact scores (see Figure 4) were significantly lower (*p* < 0.001, each) for 120-kVp-like images (median score: 2, range: 1–5) compared with 40-keV (median score: 4, range: 1–5), 190-keV (median score: 3, range: 2–5), VNC (median score: 3, range: 2–5), and iodine (median score: 4, range: 1–5) images.
(2) Twin-bean scanner: Qualitative artifact scores were significantly lower (*p* < 0.001, each) for 120-kVp-like images (median score: 2, range: 1–4) compared with 40-keV (median score: 5, range: 1–5), 190-keV (median score: 3, range: 2–5), VNC (median score: 3, range: 1–5), and iodine (median score: 4, range: 1–5) images.
(3) Fast kV-switching scanner: Qualitative artifact scores were significantly lower (*p* < 0.001, each) for iodine images (median score: 2, range: 1–5) compared with 120-kVp-like (median score: 3, range: 1–5), 40-keV (median score: 3, range: 1–5), 140-keV (median score: 3, range: 1–5), and VNC (median score: 3, range: 1–5) images.
(4) Dual-layer spectral detector scanner: Qualitative artifact scores were significantly lower (*p* < 0.001, each) for iodine images (median score: 1, range: 1–3) compared with 120-kVp (median score: 3, range: 1–5), 40-keV (median score: 3, range: 1–5), 200-keV (median score: 3, range: 2–5), and VNC (median score: 3, range: 1–5) images. Further details on qualitative artifact scores are provided in the Supplementary Material.

## 4. Discussion

We found that, in routine clinical DECT scans of the abdomen, visceral-motion-related artifacts on the liver could be minimized by viewing specific DECT image reconstructions, depending on the type of DECT scanner. For dual-source and twin-beam DECT scanners, visceral-motion-related artifacts were significantly less severe, both quantitatively and qualitatively, when the liver was viewed using 120-kVp-like images, but not iodine images. Conversely, for fast kV-switching and dual-layer spectral detector DECT scanners, visceral-motion-related artifact on the liver were significantly less severe in iodine images, but not in 120-kVp(-like) images. Dual-source 120-kVp-like images and dual-layer spectral detector iodine images reduced visceral-motion-related artifacts to an extent that no significant difference in measurements of HU or iodine concentrations between artifacts and unaffected liver parenchyma was detectable.

**Figure 5:**
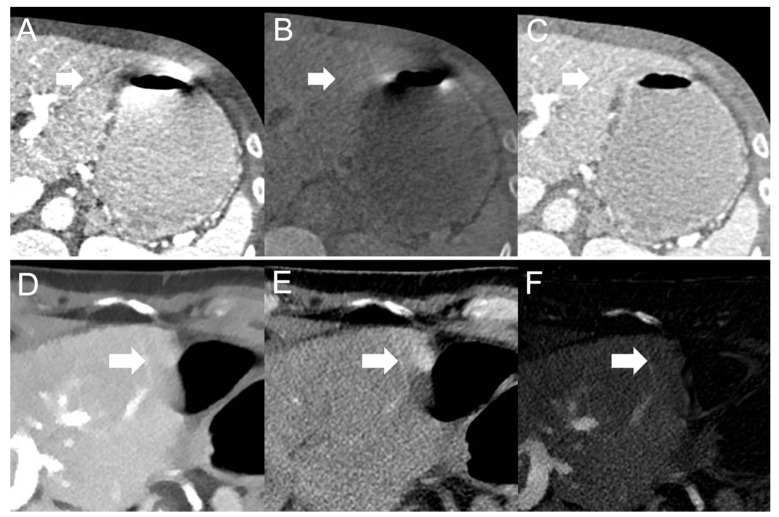
Visceral-motion-related artifact (arrows) appearance on images with (**A**,**D**) low X-ray photon energy and (**B**,**E**) high X-ray photon energy acquired with a dual-source (top row) and dual-layer spectral detector (bottom row) DECT scanner. As hyperdense artifact components on (**A**) low-energy dual-source images appear hypodense on (**B**) high-energy dual-source images, and vice versa, visceral-motion-related artifacts are substantially reduced on (**C**) blended 120-kVp-like dual-source images. As hyperdense artifact components on (**D**) low-energy dual-layer spectral detector images remain hyperdense on (**E**) high-energy dual-layer spectral detector images, and vice versa, visceral-motion-related artifacts are substantially reduced on (**F**) iodine dual-layer spectral detector images.

These strikingly different findings for different DECT implementations likely result from different gantry geometry in different DECT scanner models. Dual-source and twin-beam models acquire the high- and low-kVp datasets at dissimilar gantry positions [19]. Consequently, visceral-motion-related artifacts appear in different positions on the high- versus low-kVp datasets [29]. As the 120-kVp-like images are a blended mix of the high- and low-kVp datasets, bright and dark visceral-motion-related artifact components cancel each other out (see Figure 5). Conversely, for fast kV-switching scanners and dual-layer spectral detector scanners, there is close or perfect geometric alignment and temporal resolution of high- and low-kVp datasets [19,29]. Therefore, visceral-motion-related artifacts on these scanners have a very similar configuration of bright and dark components on the high- and low-kVp datasets. Blending high- and low-kVp datasets to reduce artifacts is not efficient in these scanner models. However, because visceral-motion-related artifacts affect the high- and low-kVp datasets to a similar degree, and iodine images depict voxels with a relatively large decrease in HU between low-kVp and high-kVp images, visceral-motion-related artifacts are substantially reduced in iodine images [9,13,29].

Our results are in line with previous studies evaluating visceral-motion-related artifact reduction in DECT image reconstructions. Winklhofer et al. showed in DECT scans of a bowel peristalsis phantom model and 100 patients acquired on a fast kV-switching scanner that general visceral-motion-related artifacts were substantially reduced in iodine images compared to 70-keV, 120-keV, and VNC images (*p* < 0.001, each) [7]. However, this study did not compare different DECT scanner models and did not further investigate the effects of visceral-motion-related artifacts on the liver, other than frequency [7]. In a phantom study Obmann et al. assessed the influence of different DECT scanner models on the severity of visceral-motion-related artifacts in 120-kVp-like and iodine images using a qualitative three-point score [29]. It was shown that visceral-motion-related artifacts were perceived as least severe in iodine images for fast kV-switching and dual-layer spectral detector scanners, and in 120-kVp-like images for dual-source and twin-beam scanners [29]. Our study adds to the field by evaluating the influence of different DECT scanner models on the severity of visceral-motion-related artifacts on the liver in actual clinical DECT scans of 458 consecutive patients. In addition to qualitative five-point artifact scores based on oncologic liver evaluation, we also obtained quantitative measurements of visceral-motion-related artifacts on the liver. Besides 120-kVp and iodine images, monoenergetic low- and high-keV images as well as VNC images were also assessed for the four commonly used DECT models.

Our study has limitations. As the appearances of the monoenergetic, VNC, and iodine images are very characteristic, effective blinding of the readers to the different image reconstructions was not possible. The four DECT techniques were not employed among the same patients, since routine clinical DECT scans were evaluated. In order to obtain the maximum effect of low-keV monoenergetic reconstructions on visceral-motion-related artifacts, monoenergetic 40-keV images were evaluated instead of more commonly used 50-keV images. Diagnostic accuracy of liver lesion detection was not assessed, as our study population did not contain a sufficient number of liver lesions masked by visceral-motion-related artifacts.

In conclusion, visceral-motion-related artifacts on the liver can be substantially reduced by viewing 120-kVp-like images for dual-source and twin-beam DECT scanners, and iodine images for fast kV-switching and dual-layer spectral detector DECT scanners.

## Figures and Tables

**Table 1:** Scan parameters of the different CT scanners. ASIR-V (adaptive statistical iterative reconstruction). ADMIRE (advanced modeled iterative reconstruction).

	Dual-Source	Twin-Beam	Fast kV-Switching	Dual-Layer Spectral Detector
Tube voltage	Source A: 100 kVp Source B: 140 kVp	120 kVp	80 and 140 kVp (0.25 millisecond kV-switching)	120 kVp
Filtration	Tin filter (Source B)	Tin/gold split-filter	none	none
Tube current–time product reference values	70 mAs (automatic tube current adaption)	70 mAs (automatic tube current adaption)	70 mAs (automatic tube current adaption)	70 mAs (automatic tube current adaption)
Collimation	2 × 64 × 0.6 mm	64 × 0.6 mm	128 × 0.625 mm	64 × 0.625 mm
Reconstruction algorithm	ADMIRE strength 3	ADMIRE strength 3	30% ASIR- V	Spectral level 3
Slice thickness	2.5 mm	2.5 mm	2.5 mm	2.5 mm
120-kVp-like images	Mix of 100 kVp and 140 kVp (tin filter)	Mix of 120 kVp (gold filter) and 120 kVp (tin filter)	70 keV	120 kVp

**Table 2:** Quantitative measurements of visceral-motion-related artifacts on the liver in 120-kVp(-like), low- and high-keV, VNC, and iodine images acquired on a dual-source, twin-beam, fast kV-switching, and dual-layer spectral detector DECT scanner. ROImax (regions of interest measurement in the most visible area and on the low-keV bright area of the artifact). ROImin (regions of interest measurement in the most visible area and on the low-keV dark area of the artifact). ROIref (regions of interest measurement in neighboring, unaffected liver parenchyma).

**Dual-source**					
**Image reconstructions**	**Quantitative artifact measurements**			***p*-values**	
	**mean ROImax**	**mean ROImin**	**mean ROIref**	**mean ROImax compared with mean ROIref**	**mean ROImin compared with mean ROIref**
120-like	97 HU	105 HU	108 HU	*p* < 0.001	*p* = 0.32
40 keV	345 HU	39 HU	277 HU	*p* < 0.001	*p* < 0.001
190 keV	36 HU	126 HU	68 HU	*p* < 0.001	*p* < 0.001
VNC	35 HU	107 HU	62 HU	*p* < 0.001	*p* < 0.001
Iodine	4.0 mg/mL	−2.7 mg/mL	2.5 mg/mL	*p* < 0.001	*p* < 0.001
**Twin-beam**					
**Image reconstructions**	**Quantitative artifact measurements**			***p*-values**	
	**mean ROImax**	**mean ROImin**	**mean ROIref**	**mean ROImax compared with mean ROIref**	**mean ROImin compared with mean ROIref**
120-like	98 HU	101 HU	106 HU	*p* = 0.006	*p* = 0.03
40 keV	513 HU	−79 HU	269 HU	*p* < 0.001	*p* < 0.001
190 keV	21 HU	142 HU	70 HU	*p* < 0.001	*p* < 0.001
VNC	25 HU	107 HU	67 HU	*p* < 0.001	*p* < 0.001
Iodine	4.8 mg/mL	−3.1 mg/mL	2.3 mg/mL	*p* < 0.001	*p* < 0.001
**Fast kV-switching**					
**Image reconstructions**	**Quantitative artifact measurements**			***p*-values**	
	**mean ROImax**	**mean ROImin**	**mean ROIref**	**mean ROImax compared with mean ROIref**	**mean ROImin compared with mean ROIref**
120-like	183 HU	55 HU	115 HU	*p* < 0.001	*p* < 0.001
40 keV	379 HU	151 HU	255 HU	*p* < 0.001	*p* < 0.001
140 keV	106 HU	19 HU	62 HU	*p* < 0.001	*p* < 0.001
VNC	94 HU	21 HU	59 HU	*p* < 0.001	*p* < 0.001
Iodine	3.8 mg/mL	1.8 mg/mL	2.6 mg/mL	*p* < 0.001	*p* < 0.001
**Dual-layer spectral detector**					
**Image reconstructions**	**Quantitative artifact measurements**			***p*-values**	
	**mean ROImax**	**mean ROImin**	**mean ROIref**	**mean ROImax compared with mean ROIref**	**mean ROImin compared with mean ROIref**
120 kVp	134 HU	86 HU	102 HU	*p* < 0.001	*p* < 0.001
40 keV	242 HU	173 HU	216 HU	*p* < 0.001	*p* < 0.001
200 keV	94 HU	54 HU	62 HU	*p* < 0.001	*p* < 0.001
VNC	89 HU	50 HU	74 HU	*p* < 0.001	*p* < 0.001
Iodine	1.9 mg/mL	1.7 mg/mL	2.1 mg/mL	*p* = 0.15	*p* < 0.001

## Data Availability

Data are available upon reasonable request. Requests should be sent to the corresponding author and are subject to approval.

## Supplementary Materials

The following supporting information can be downloaded at: https://www.mdpi.com/article/10.3390/diagnostics12092155/s1. Materials and Methods S: CT image acquisition. Results S: Study population. Figure S1: Depth of extension of visceral-motion-related artifacts into the liver parenchyma. Measurements of Reader A only. Figure S2: Depth of extension of visceral-motion-related artifacts into the liver parenchyma. Measurements of Reader B only. Figure S3: Qualitative artifact scores of Reader A only. Figure S4: Qualitative artifact scores of Reader B only. Table S1: Quantitative measurements of visceral-motion-related artifacts on the liver of Reader A only. Table S2: Quantitative measurements of visceral-motion-related artifacts on the liver of Reader B only.

## Abbreviations

Computed tomography (CT), DECT (Dual-energy computed tomography), HU (Hounsfield Units), intraclass correlation coefficients (ICC), regions of interest (ROI), standard deviation (SD), virtual non-contrast (VNC).
