# Supplementary material for: Reducing Visceral-Motion-Related Artifacts on the Liver with Dual-Energy CT: A Comparison of Four Different CT Scanner Techniques
Source: Diagnostics (Basel). 2022 Sep 5;12(9):2155. doi: 10.3390/diagnostics12092155 (PMC9497818; doi:10.3390/diagnostics12092155)
Supplement: Supplementary file 1 [file diagnostics-12-02155-s001.zip › diagnostics-1890126-supplementary.pdf]

Reducing Visceral-Motion-Related Artifacts on the Liver with Dual-Energy CT: A Comparison of Four Different CT Scanner Techniques

Supplemental Material

Materials and Methods

CT image acquisition

Dual-energy CT image data sets were acquired on four different dual-energy CT scanners:

1) Dual-source CT scanner (Somatom Definition Flash; Siemens Healthineers, Forchheim, Germany) in helical scanning mode, axial acquisition plane, collimation of 0.6 mm. Dual-energy data were acquired using two X-ray tubes with an angular offset of 95° at tube voltages of 140 kVp (tin filter) and 100 kVp, respectively, tube current–time product reference values of 70 mAs and automatic tube current adaption.

2) Twin-beam CT scanner (Somatom Definition Edge; Siemens Healthineers, Forchheim, Germany) in helical scanning mode, axial acquisition plane, collimation of 0.6 mm. Dual-energy data were acquired using a tin/gold split-filter twin-beam at a tube voltage of 120 kVp, tube current–time product reference values of 70 mAs and automatic tube current adaption.

3) Fast kV-switching CT scanner (Revolution; GE Healthcare, Chicago, Illinois) in helical scanning mode, 0.625 mm collimation. Dual-energy data were acquired with 0.25 millisecond kV-switching between 80 kVp and 140 kVp tube voltage at tube current–time product reference values of 70 mAs using automatic tube current adaption.

4) Dual-layer spectral detector CT scanner (IQon; Philips Healthcare, Cleveland, Ohio) in helical scanning mode, axial acquisition plane, collimation of 0.625 mm. Tube voltage was 120 kVp at tube current–time product reference values of 70 mAs using automatic tube current adaption. Dual-energy data were acquired on a dual-layer spectral detector with a top-layer yttrium-based garnet scintillator and bottom-layer gadolinium oxysulphide scintillator.

Results

Study population

1) Dual-source scanner: 127 CT scans of 90 consecutive patients (mean age: 58±14 years, 50 men) were performed on the dual-source scanner. 10/127 (8%) CT scans were excluded due to artifacts on the liver originating from metallic foreign materials. Visceral motion-related artifacts on the liver were present in 43/117 (37%) CT scans.

2) Twin-beam scanner: 142 CT scans of 140 consecutive patients (mean age: 51±19 years, 72 men) were performed on the twin-bean scanner. 11/142 (8%) CT scans were excluded due to artifacts on the liver originating from metallic foreign materials. Visceral motion-related artifacts on the liver were present in 38/131 (29%) CT scans.

3) Fast kV-switching scanner: 126 CT scans of 97 consecutive patients (mean age: 66±11 years, 89 men) were performed on the fast kV-switching scanner. 4/126 (3%) CT scans were excluded due to artifacts on the liver originating from metallic foreign materials. Visceral motion-related artifacts on the liver were present in 45/122 (37%) CT scans. 10/45 (22%) CT scans with visceral motion-related artifacts on the liver were excluded from further evaluation due to missing source dual-energy data.

4) Dual-layer spectral detector scanner: 220 CT scans of 131 consecutive patients (mean age: 68±10 years, 120 men) were performed on the dual-layer spectral detector scanner. 10/220 (5%) CT scans were excluded due to artifacts on the liver originating from metallic foreign materials, 1/220 (1%) CT scan was excluded due to artifacts on the liver originating from oral contrast material. Visceral motion-related artifacts on the liver were present in 52/209 (25%) CT scans. 1/52 (2%) CT scan with visceral motion-related artifacts on the liver was excluded from further evaluation due to missing source dual-energy data.

Supplementary Figure S1

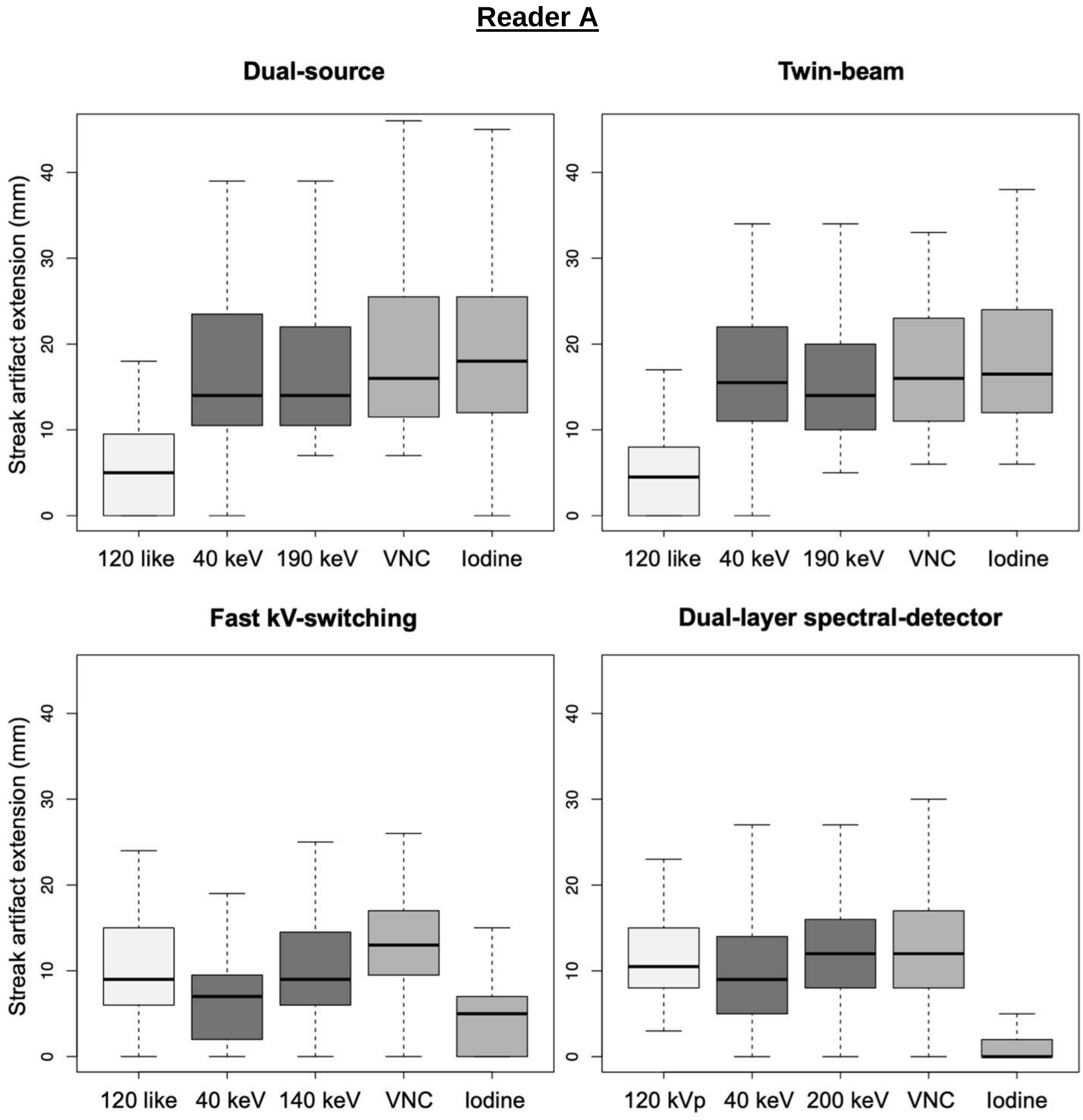

Figure S1: Depth of extension of visceral motion-related artifacts into the liver parenchyma measured in axial 120 kVp (like), low and high keV, VNC, and iodine images acquired on a dual-source, twin-beam, fast kV-switching, and dual-layer spectral detector CT scanner. Measurements of **Reader A** only.

Supplementary Figure S2

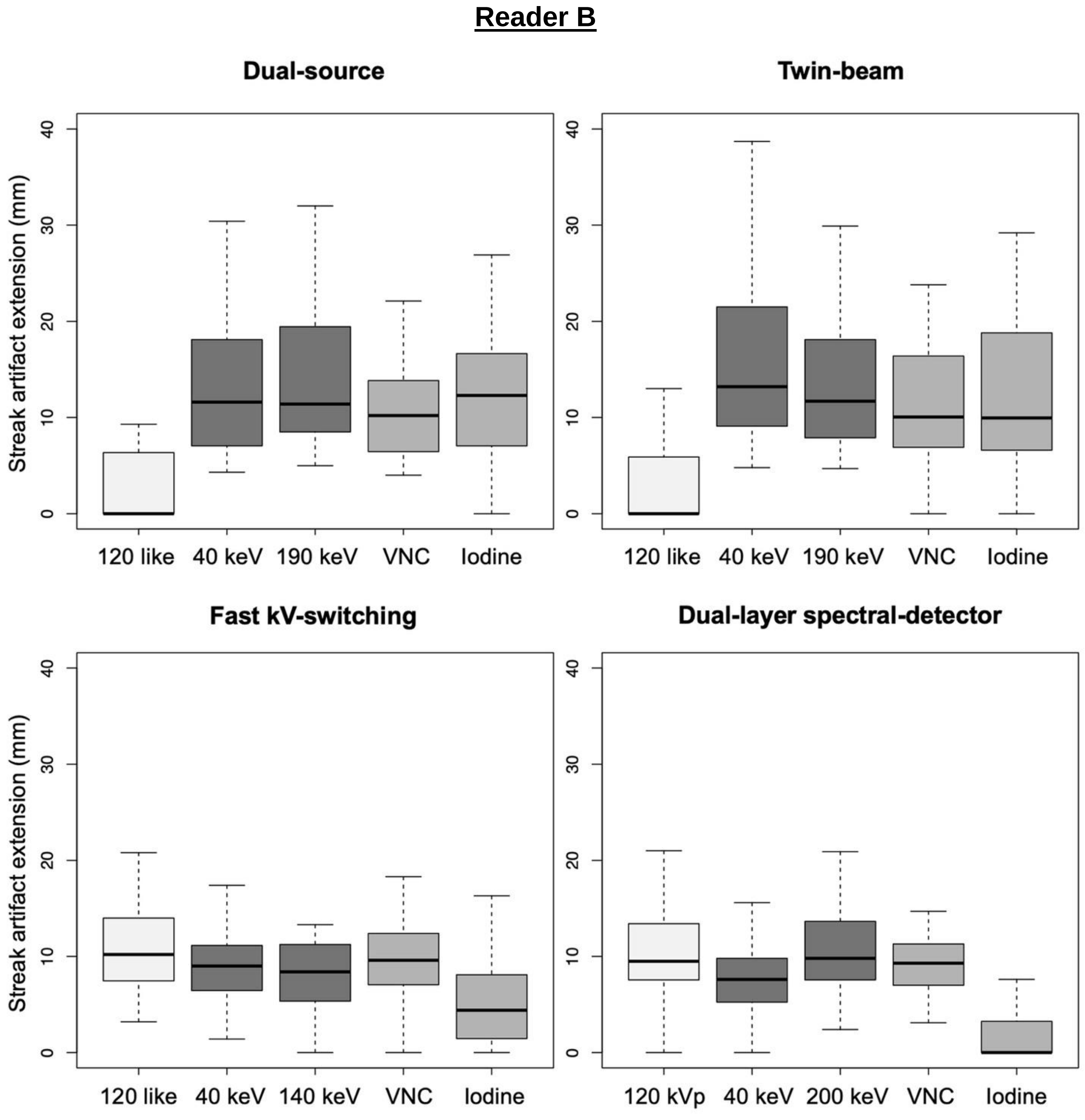

Figure S2: Depth of extension of visceral motion-related artifacts into the liver parenchyma measured in axial 120 kVp (like), low and high keV, VNC, and iodine images acquired on a dual-source, twin-beam, fast kV-switching, and dual-layer spectral detector CT scanner. Measurements of **Reader B** only.

Supplementary Figure S3

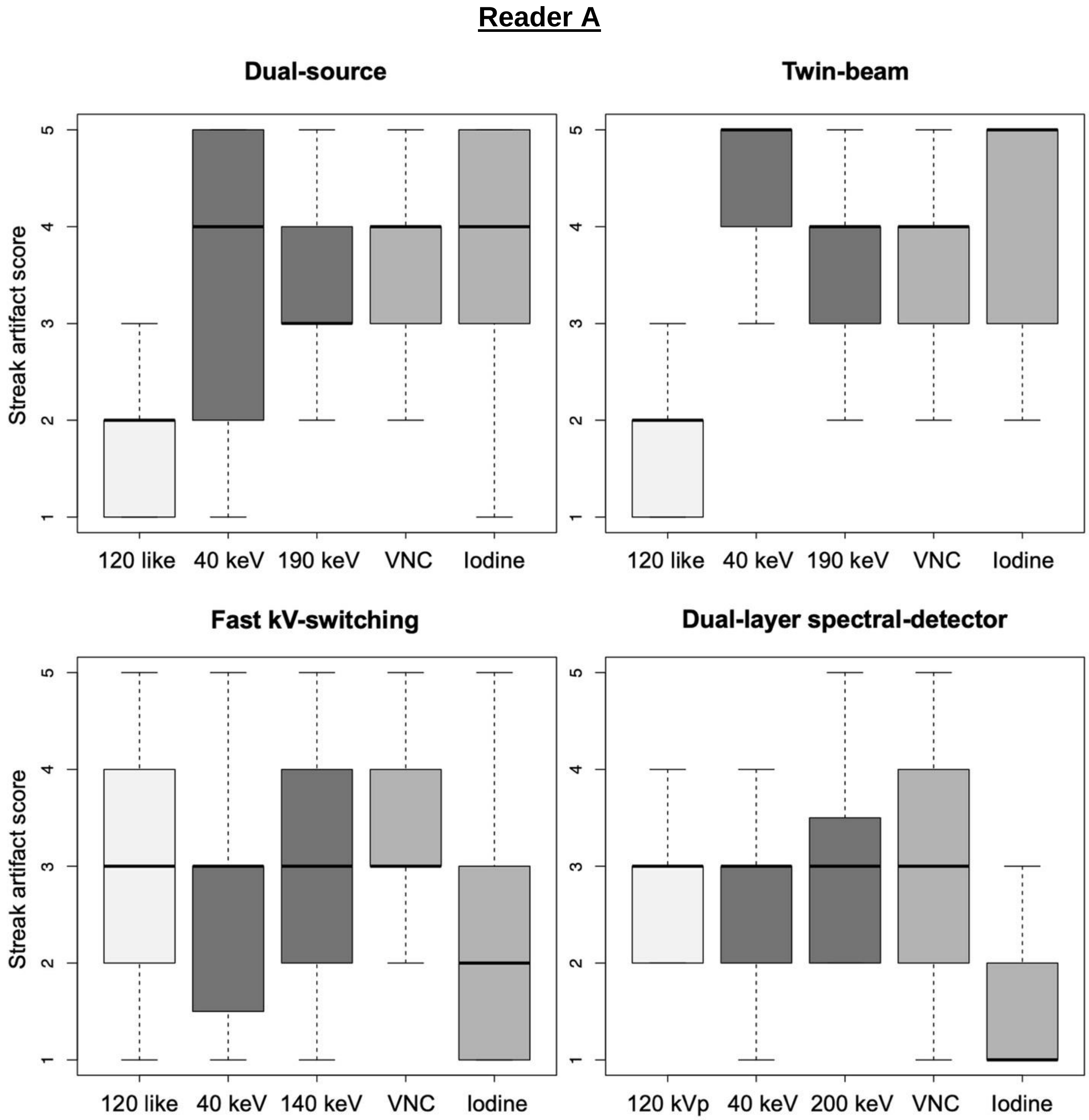

Figure S3: Qualitative artifact scores (1=none to 5=severe) of visceral motion-related artifacts on the liver in axial 120 kVp (like), low keV, high keV, VNC, and iodine images acquired on a dual-source, twin-beam, fast kV-switching, and dual-layer spectral detector CT scanner. Scores of **Reader A** only.

Supplementary Figure S4

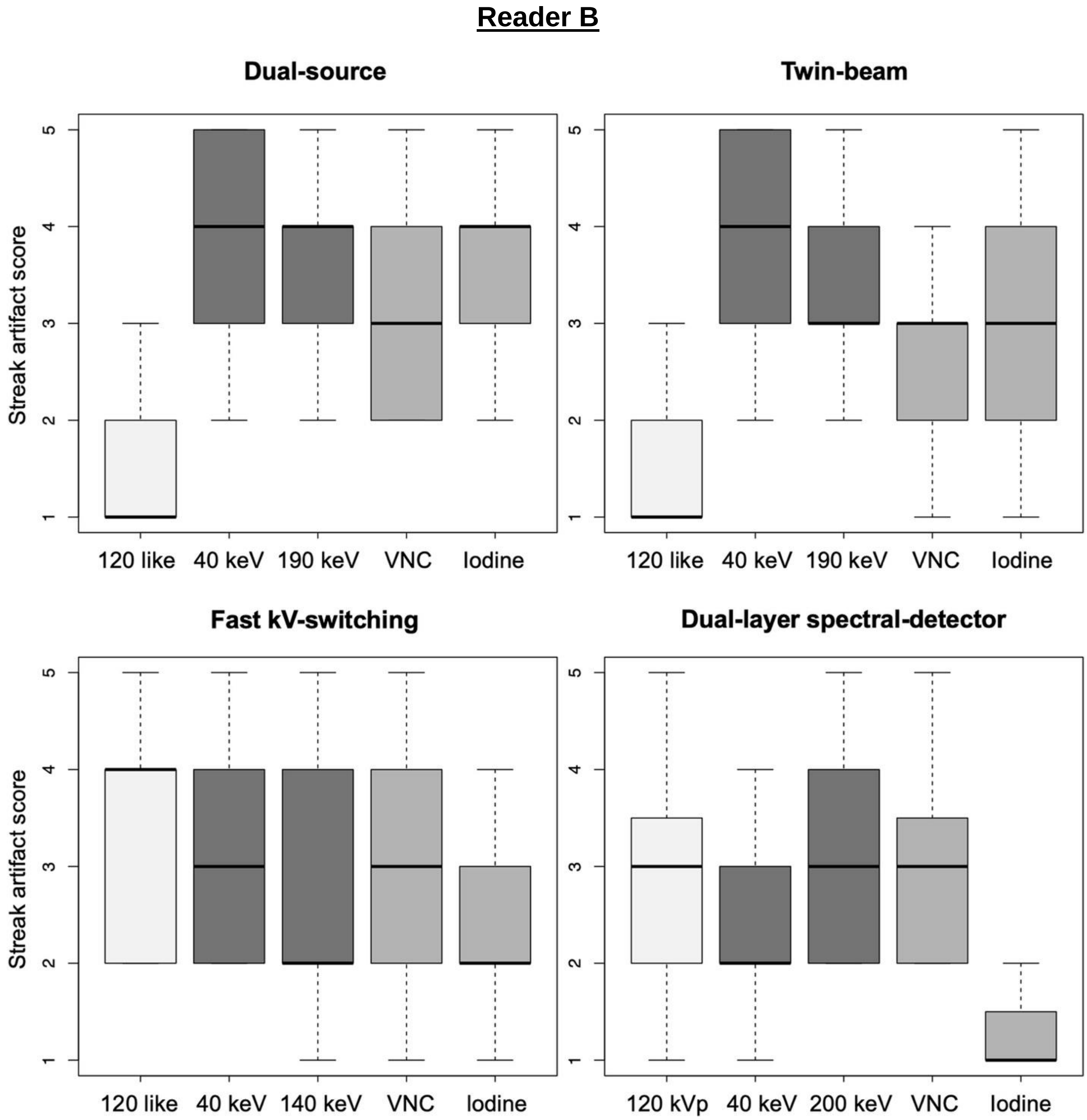

Figure S4: Qualitative artifact scores (1=none to 5=severe) of visceral motion-related artifacts on the liver in axial 120 kVp (like), low keV, high keV, VNC, and iodine images acquired on a dual-source, twin-beam, fast kV-switching, and dual-layer spectral detector CT scanner. Scores of **Reader B** only.

Table S1

Dual-source (Reader A)					
Image reconstructions	Quantitative artifact measurements			p-values	
	**mean ROImax**	**mean ROImin**	**mean ROIref**	mean ROImax compared with mean ROIref	mean ROImin compared with mean ROIref
**120 like**	99 HU	105 HU	110 HU	p<0.001	**p=0.09**
**40 keV**	317 HU	148 HU	282 HU	**p=0.11**	p<0.001
**190 keV**	42 HU	96 HU	68 HU	p<0.001	p<0.001
**VNC**	35 HU	101 HU	64 HU	p<0.001	p<0.001
**Iodine**	4.0 mg/ml	-1.6 mg/ml	2.5 mg/ml	p=0.01	p<0.001

Twin-beam (Reader A)					
Image reconstructions	Quantitative artifact measurements			p-values	
	**mean ROImax**	**mean ROImin**	**mean ROIref**	mean ROImax compared with mean ROIref	mean ROImin compared with mean ROIref
**120 like**	97 HU	101 HU	107 HU	p=0.01	**p=0.11**
**40 keV**	420 HU	75 HU	262 HU	p<0.001	p=0.001
**190 keV**	24 HU	101 HU	70 HU	p<0.001	p=0.03
**VNC**	16 HU	106 HU	70 HU	p<0.001	p<0.001
**Iodine**	5.2 mg/ml	-1.9 mg/ml	2.1 mg/ml	p<0.001	p<0.001

Fast kV-switching (Reader A)					
Image reconstructions	Quantitative artifact measurements			p-values	
	**mean ROImax**	**mean ROImin**	**mean ROIref**	mean ROImax compared with mean ROIref	mean ROImin compared with mean ROIref
**120 like**	172 HU	61 HU	117 HU	p<0.001	p<0.001
**40 keV**	343 HU	161 HU	248 HU	p<0.001	p<0.001
**140 keV**	103 HU	20 HU	62 HU	p<0.001	p<0.001
**VNC**	92 HU	23 HU	59 HU	p<0.001	p<0.001
**Iodine**	3.3 mg/ml	1.9 mg/ml	2.62 mg/ml	p<0.001	p<0.001

Dual-layer spectral detector (Reader A)					
Image reconstructions	Quantitative artifact measurements			p-values	
	**mean ROImax**	**mean ROImin**	**mean ROIref**	mean ROImax compared with mean ROIref	mean ROImin compared with mean ROIref
**120 kVp**	125 HU	82 HU	100 HU	p<0.001	p<0.001
**40 keV**	226 HU	173 HU	201 HU	p<0.001	p<0.001
**200 keV**	89 HU	50 HU	68 HU	p<0.001	p<0.001
**VNC**	84 HU	46 HU	61 HU	p<0.001	p<0.001
**Iodine**	1.7 mg/ml	1.5 mg/ml	1.7 mg/ml	**p=0.74**	p<0.001

Table S1: Quantitative measurements of visceral motion-related artifacts on the liver on 120 kVp (like), low keV, high keV, VNC, and iodine images acquired on a dual-source, twin-beam, fast

kV-switching, and dual-layer spectral detector DECT scanner. ROImax (regions of interest measurement in the most visibly on low keV bright area of the artifact). ROImin (regions of interest measurement in the most visibly on low keV dark area of the artifact). ROIref (regions of interest measurement in the neighboring liver parenchyma not affected by artifacts). Measurements of **Reader A** only.

Table S2

Dual-source (Reader B)					
Image reconstructions	Quantitative artifact measurements			p-values	
	**mean ROImax**	**mean ROImin**	**mean ROIref**	mean ROImax compared with mean ROIref	mean ROImin compared with mean ROIref
**120 like**	95 HU	105 HU	106 HU	p=0.01	**p=0.87**
**40 keV**	373 HU	-70 HU	272 HU	p<0.001	p<0.001
**190 keV**	30 HU	156 HU	68 HU	p<0.001	p<0.001
**VNC**	34 HU	113 HU	60 HU	p<0.001	p<0.001
**Iodine**	4.0 mg/ml	-3.7 mg/ml	2.6 mg/ml	p=0.005	p<0.001

Twin-beam (Reader B)					
Image reconstructions	Quantitative artifact measurements			p-values	
	**mean ROImax**	**mean ROImin**	**mean ROIref**	mean ROImax compared with mean ROIref	mean ROImin compared with mean ROIref
**120 like**	99 HU	101 HU	105 HU	**p=0.09**	**p=0.16**
**40 keV**	607 HU	-232 HU	276 HU	p=0.008	p=0.001
**190 keV**	18 HU	183 HU	70 HU	p<0.001	p=0.03
**VNC**	33 HU	108 HU	64 HU	p<0.001	p<0.001
**Iodine**	4.3 mg/ml	-4.3 mg/ml	2.5 mg/ml	p=0.005	p<0.001

**Fast kV-switching (Reader B)**					
Image reconstructions	Quantitative artifact measurements			p-values	
	**mean ROImax**	**mean ROImin**	**mean ROIref**	mean ROImax compared with mean ROIref	mean ROImin compared with mean ROIref
**120 like**	195 HU	50 HU	114 HU	p<0.001	p<0.001
**40 keV**	415 HU	142 HU	262 HU	p<0.001	p<0.001
**140 keV**	108 HU	18 HU	61 HU	p<0.001	p<0.001
**VNC**	96 HU	20 HU	58 HU	p<0.001	p<0.001
**Iodine**	4.0 mg/ml	1.7 mg/ml	2.5 mg/ml	p<0.001	p<0.001

**Dual-layer spectral detector (Reader B)**					
Image reconstructions	Quantitative artifact measurements			p-values	
	**mean ROImax**	**mean ROImin**	**mean ROIref**	mean ROImax compared with mean ROIref	mean ROImin compared with mean ROIref
**120 kVp**	143 HU	65 HU	99 HU	p<0.001	p<0.001
**40 keV**	252 HU	144 HU	196 HU	p<0.001	p<0.001
**200 keV**	101 HU	37 HU	64 HU	p<0.001	p<0.001
**VNC**	97 HU	33 HU	59 HU	p<0.001	p<0.001
**Iodine**	1.8 mg/ml	1.3 mg/ml	1.7 mg/ml	p<0.001	p<0.001

Table S2: Quantitative measurements of visceral motion-related artifacts on the liver on 120 kVp (like), low keV, high keV, VNC, and iodine images acquired on a dual-source, twin-beam, fast

kV-switching, and dual-layer spectral detector DECT scanner. ROImax (regions of interest measurement in the most visibly and on low keV bright area of the artifact). ROImin (regions of interest measurement in the most visibly and on low keV dark area of the artifact). ROIref (regions of interest measurement in the neighboring liver parenchyma not affected by artifacts). Measurements of **Reader B** only.
